# Metalloporphyrin Pd(T4) Exhibits Oncolytic Activity and Cumulative Effects with 5-ALA Photodynamic Treatment against C918 Cells

**DOI:** 10.3390/ijms21020669

**Published:** 2020-01-20

**Authors:** Brandon Leviskas, Tibor Valyi-Nagy, Gnanasekar Munirathinam, Matthew Bork, Klara Valyi-Nagy, Troy Skwor

**Affiliations:** 1 Department of Chemical and Biological Sciences, Rockford University, Rockford, IL 61107, USA; blevis1@lsu.edu (B.L.); mbork@rockford.edu (M.B.); 2 Department of Pathology, College of Medicine, University of Illinois at Chicago, Chicago, IL 60612, USA; tiborv@uic.edu (T.V.-N.); klaravn@uic.edu (K.V.-N.); 3 Department of Biomedical Sciences, College of Medicine at Rockford, University of Illinois, Rockford, IL 61108, USA; mgnanas@uic.edu; 4 Department of Biomedical Sciences, University of Wisconsin—Milwaukee, Milwaukee, WI 53211, USA

**Keywords:** Pd(T4), melanoma, reactive oxygen species, 5-ALA, blue light, apoptosis, necrosis

## Abstract

Photodynamic therapy is a non-invasive method where light activates a photosensitizer bound to cancer cells, generating reactive oxygen species and resulting in cell death. This study assessed the oncolytic potential of photodynamic therapy, comparing European Medicines Agency and United States Food and Drug Administration-approved 5-aminolevulinic acid (5-ALA) to a metalloporphyrin, Pd(T4), against a highly invasive uveal melanoma cell line (C918) in two- and three-dimensional models *in vitro*. Epithelial monolayer studies displayed strong oncolytic effects (>70%) when utilizing Pd(T4) at a fraction of the concentration, and reduced pre-illumination time compared to 5-ALA post-405 nm irradiance. When analyzed at sub-optimal concentrations, application of Pd(T4) and 5-ALA with 405 nm displayed cumulative effects. Lethality from Pd(T4)-photodynamic therapy was maintained within a three-dimensional model, including the more resilient vasculogenic mimicry-forming cells, though at lower rates. At high concentrations, modality of cell death exhibited necrosis partially dependent on reactive oxygen species. However, sub-optimal concentrations of photosensitizer exhibited an apoptotic protein expression profile characterized by increased Bax/Bcl-2 ratio and endoplasmic stress-related proteins, along with downregulation of apoptotic inhibitors CIAP-1 and -2. Together, our results indicate Pd(T4) as a strong photosensitizer alone and in combination with 5-ALA against C918 cells.

## 1. Introduction

Current cancer treatments vary depending on stage and location, and can include surgical excision, radiation therapy, chemotherapy, immunotherapy, hormone therapy, and combinatorial therapy. Each option, while being effective for some, still leaves many patients with adverse side effects. Despite removal at the site of detection, cancer can reappear months to years later in the vicinity and elsewhere in the body [1]. Regarding chemotherapy and immunotherapy, each may be effective modalities of treatment, but potentially leave patients with toxic outcomes or establishment of drug resistance [2,3,4]. These toxicities can include neurotoxicity, cardiotoxicity, immunological complications, rash, hepatitis, and sarcopenia, among others [5,6]. Although radiation therapy may work for many patients, the type of cancer, genetic mutations, and age of the patient may limit any significant protective effects [7], as well as increase the risk for the development of a second primary cancer [8].

Photodynamic therapy (PDT) utilizes light juxtaposed with a photosensitizer in the presence of oxygen, resulting in potentially cytolytic effects due to reactive oxygen species (ROS) production. One well-studied European Medicines Agency (EMA) and United States Food and Drug Administration (FDA) approved photosensitizer, 5-aminolevulinic acid (5-ALA), is utilized for various clinical PDT applications [9,10], including actinic keratosis [11,12] and basal cell carcinoma [13], as well as its use as a surgical guidance tool for glioblastomas [14,15] and gliomas [16]. Intrinsically, 5-ALA is a heme precursor in a metabolic process within the mitochondria that synthesizes protoporphyrin IX (PPIX) [17,18]. Some caveats of the usage of 5-ALA-inducedPPIX is the delay in synthesis following administration, the targeting capability and delivery of 5-ALA being reliant on accumulation, and active mitochondrial membrane transport regulated through altered protein expression. ROS production from ALA in vitro [19,20,21] and in vivo [18,22,23,24] occurs at multiple wavelengths of excitation, ranging from a blue spectrum with a soret peak at 397 nm, to a red spectrum Q band at 670 nm [25,26].

Our study analyzed oncolytic effects of the metalloporphyrin Pd(T4) compared to the current FDA-approved 5-ALA in two- and three-dimensional cell cultures with blue light. Photosensitizer Pd(T4) has demonstrated elevated singlet oxygen production post-illumination compared to its base conjugate TMPyP [27], resulting in photodynamic inactivation of bacteria such as methicillin-resistant *Staphylococcus aureus* and *Escherichia coli* [27]. TMPyP4, the base conjugate, has exhibited a strong affinity for G4 quadruplexes [28,29,30], as well as other macromolecules [31,32]. Our study assessed the oncolytic potential of Pd(T4) when used in a photodynamic system as a primary or potential combinatorial component with 5-ALA and blue light against cancer cells.

## 2. Results

### 2.1. Oncolytic Effects of Photosensitizer Pd(T4) Compared to 5-ALA during PDT

**Figure 1:**
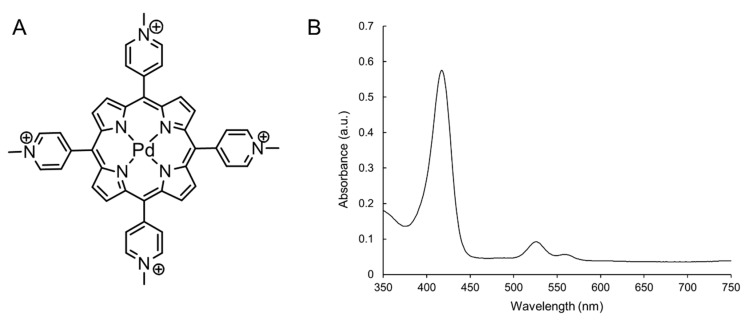
Characteristics of Pd(T4). Structure (**A**) and absorbance spectrum (**B**) of Pd(T4).

In numerous countries including the United States, 5-ALA has been approved for treatment against an array of clinical anomalies, including actinic keratosis and basal cell carcinoma, using either blue or red light [33,34]. Our study compared oncolytic activity of Pd(T4) (Figure 1) and 5-ALA after 405 nm irradiation against the highly aggressive and invasive melanoma cell line C918 [35]. Strong oncolytic activity was evident with PDT using PdT4 and 405 nm, though was inversely correlated with cell confluency. In order to test for more clinical relevance regarding direct cell–cell contact, our PDT studies were performed at 90–100% confluency.

**Figure 2:**
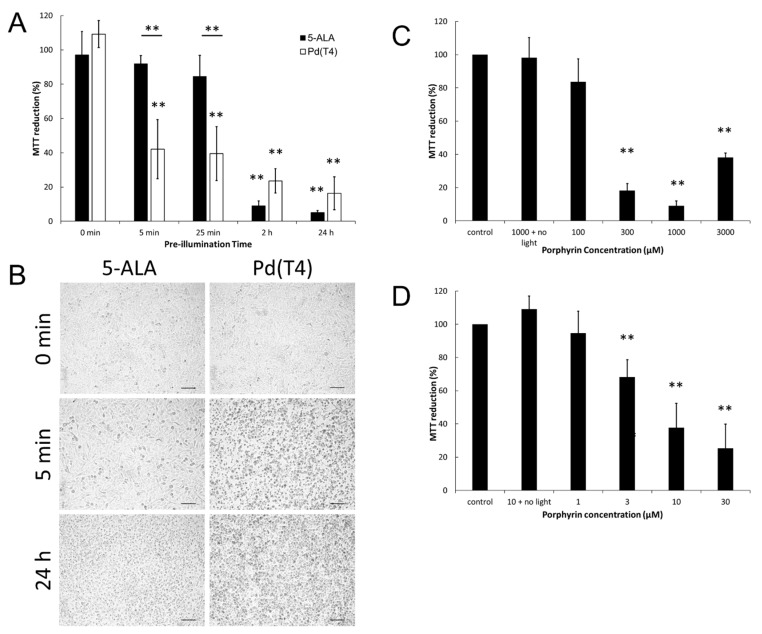
Effects of pre-illumination time and photosensitizer concentrations on oncolytic activity. C918 melanoma cells were grown to 90–100% confluency, then incubated with porphyrin 5-aminolevulinic acid (5-ALA) (1 mM) or Pd(T4) (10 µM) at various times with subsequent exposure to 60 mW of 405 nm light for 88 s (5 J/cm^2^) or without light (0 s). (**A**) Dark bars represent 5-ALA (1 mM) and open bars Pd(T4) (10 µM). Percent 3-(4,5-dimethylthiazol-2-yl)-2,5-diphenyltetrazolium bromide (MTT) reduction was determined by dividing the absorbance value from a MTT assay of the treatment group by cells alone and multiplied by 100. Data represents average ± standard deviation of duplicate wells from three to five independent experiments. (**B**) Morphological changes of cells after 24 h post-irradiance comparing the effect of photosensitizer pre-incubation at 5 min or 24 h with and without (0 s) 5 J/ cm^2^ of 405 nm irradiance. Scale bar represents 100 µm. Cells were incubated with different concentrations of photosensitizers: 5-ALA (**C**) or Pd(T4) (**D**) for 2 h before irradiance with 5 J/cm^2^ of 405 nm light. Cell viability was tested post-photodynamic therapy (PDT) using a MTT assay analyzing the percent MTT reduction by dividing the absorbance of the treatment group against cells alone multiplied by 100. Data represents average + standard deviation of duplicate wells from three to five independent experiments. Statistical differences between the absence and presence of light treatments (**A**), between treatment groups at similar time points (**A**), and against different concentrations of porphyrins (**C**,**D**) were analyzed using one-way analysis of variance (ANOVA) with Tukey’s multiple comparison test. Note: ** *p* < 0.005.

Higher healthcare costs as well as increased patient stress are associated with clinical treatment times, thus photosensitizer pre-illumination times were assessed. Minimal changes in viability were evident due to dark phototoxicity with either a photosensitizer (Figure 2A,B) or with 5 J/cm^2^ of 405 nm irradiation alone. In agreement with previous studies [18,19,20], administration of 5-ALA (1 mM) for 2 h before 5 J/cm^2^ of 405 nm light exposure showed a 91% oncolytic response (Figure 2A, *p* < 0.005), though minimal effects were evident at 25 min pre-illumination (Figure 2A,B, 91% viability). However, metalloporphyrin Pd(T4) (10 µM) diminished viability by 58% (Figure 2A, *p* < 0.05) as early as 5 min pre-illumination, followed by a fluence of 5 J/cm^2^ from a 405 nm portable light-emitting diode LED. Pd(T4) demonstrated stronger oncolytic abilities at both 5 and 25 min (Figure 2A, *p* < 0.005). No statistical differences were evident if you prolonged pre-illumination beyond 2 h with either photosensitizers, though both exhibited greater than 80% lytic activity (Figure 2A).

In Figure 2C, a narrow range of 5-ALA concentrations of 300–1000 µM demonstrated optimal lytic effects with diminishing effects at lower (100 µM) and higher (3000 µM) concentrations. Conversely, PdT4 demonstrated a different pattern where higher concentrations maintained strong oncolytic effects (Figure 2D).

### 2.2. Combinatorial Effects of Two Different Photosensitizers

**Figure 3:**
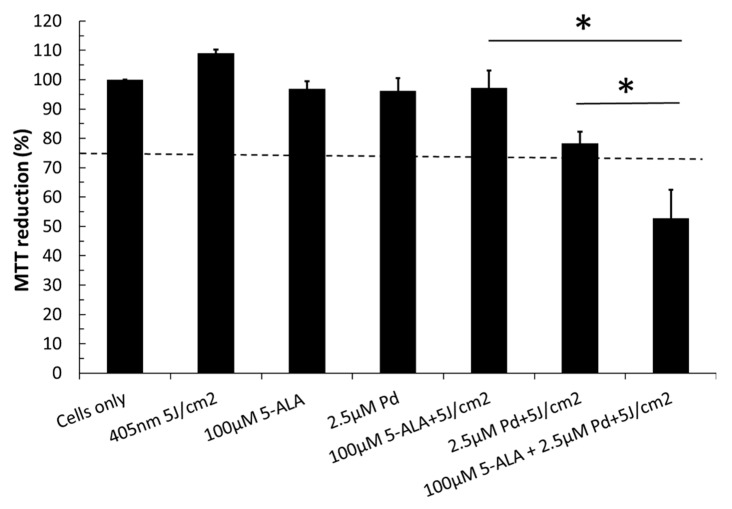
Combinatorial effects of 5-ALA and Pd(T4) on photodynamic lysis. Melanoma cells were subjected to treatment with porphyrins 5-ALA, Pd(T4), or both. Cells were grown to 90–100% confluency then incubated with either a photosensitizer or with 5-ALA ten minutes before Pd(T4). After a 2 h incubation, cells were irradiated with 5 J/cm^2^ of 405 nm LEDs. Viability was tested using MTT assay 24 h post-PDT and determined by the percent of MTT reduction, which in turn is determined by dividing the absorbance of treatment groups over cells alone. Data represents average + standard deviation of duplicate wells from three independent experiments using a one-way ANOVA with Tukey’s multiple comparison test. Statistical differences between single treatment and combinatorial treatments were represented by * *p* < 0.005. The dotted line represents where a cumulative effect between 5-ALA and Pd(T4) would exist.

Considering 5-ALA is taken up by the cells and utilized to synthesize protoporphyrin IX, which acts as the photosensitizer, and that Pd(T4) is active upon uptake, we were curious how they would work together. Sub-optimal concentrations of Pd(T4) (2.5 µM) and 5-ALA (100 µM) were treated with 5 J/cm^2^ of 405 nm, resulting in 78.2% and 97.1% viability; however, when co-administered, a cumulative effect was evident, exhibiting only 52.8% viability in C918 melanoma cells (Figure 3, *p* < 0.005).

### 2.3. PDT on Three-Dimensional Cultures with Vasculogenic Mimicry-Forming Cells

**Figure 4:**
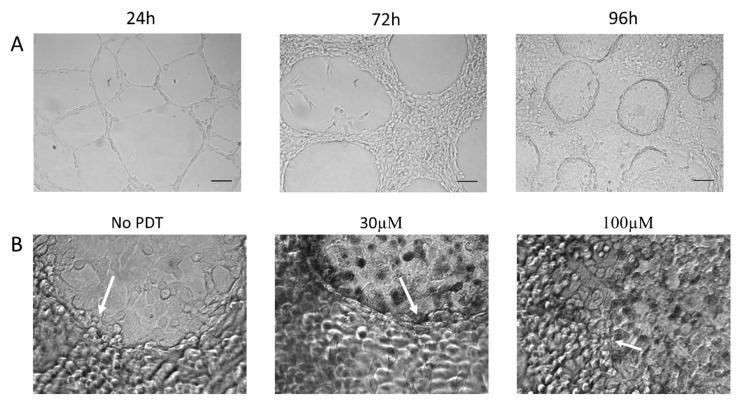
PDT on three-dimensional cultures with Pd(T4) with 405 nm irradiance. (**A**) C918 were grown on Matrigel to acquire three-dimensional cultures over 24 h, 72 h, and 96 h. Scale bar is 100 µm (**B**) Cells were grown for 120 h and left untreated or exposed to Pd(T4) at 30 µM and 100µM with subsequent 15 J/cm^2^ of 405 nm. Arrows point to vasculogenic mimicry-forming cells. Magnification 200×.

Invasion of tissue is a hallmark characteristic of aggressive cancers. Intra-tumoral heterogeneity, including the presence of cancer stem cells, have provided increased resistance to chemotherapeutic agents. In Figure 4, we tested the invasive properties of the cell line, as well as the oncolytic activity of PDT with Pd(T4) and 405 nm irradiation in a three-dimensional model exhibiting a sub-population of vasculogenic mimicry-forming, cancer stem-cell-like cells expressing marker CD271 [35]. In Figure 4A, growth is observed over the period of 96 h of C918 melanoma cells in the Matrigel system. At 24 h, some cells grow on the top surface of Matrigel, while others take on a more mesenchymal appearance, invading the Matrigel and creating a “scaffold” for further growth. By 96 h, three cell populations are evident: cells growing on the top of Matrigel, vasculogenic mimicry-forming cells displaying a honeycomb-like appearance within the Matrigel, and cells coating the bottom of the plate. Due to the increased cell population within the well and the more resilient vascular mimicry cells, both porphyrin and irradiance levels were increased. When analyzing PDT effects against C918 cells grown in the Matrigel system, cells coating the bottom of the well were most susceptible to PDT with Pd(T4) and 405 nm (Table 1). Dark phototoxicity was not evident even at porphyrin concentrations up to 100 µM (Table 1). Oncolytic activity required significantly more blue light and photosensitizers compared to two-dimensional. Although minimal PDT effects were evident at 30 µM with 10 J/cm^2^ (Table 1), drastic morphological impairment involving 50% lysis of vasculogenic mimicry-forming cells and 90% of epithelial cells residing below the Matrigel was evident with 100 µM of PdT4 and 15 J/cm^2^ of 405 nm irradiance (Figure 4B and Table 1).

### 2.4. Oncolytic Activity Mediated through Reactive Oxygen Species

**Figure 5:**
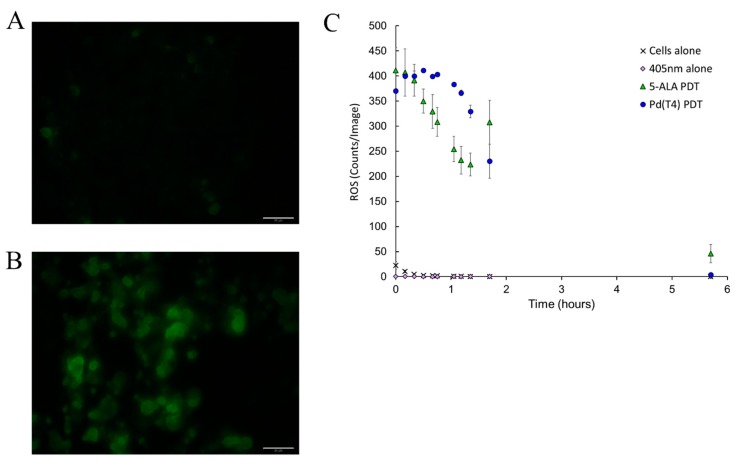
Reactive oxygen species (ROS) involvement in oncolytic activity associated with PDT. Cells were treated with 5-ALA (1 mM) or Pd(T4) (10 µM) for 2 h before 5 J/cm^2^ of irradiance with 405 nm. ROS production was visualized by 2′,7′-dichlorodihydrofluorescein diacetate (DCF) staining on cells alone (**A**) and 2 h post-PDT with Pd(T4) and 5 J/cm^2^ of 405 nm (**B**). Scale bars represent 50 µm. (**C**) The kinetic profile of ROS production post-PDT in live cells was determined using live imaging with IncuCyte^®^ and CellROX^®^ deep red probes. Results are the mean fluorescent counts ± standard deviation of four sampling within each treatment group. Note: X, cells alone; diamonds, 5 J/cm^2^ 405 nm alone; triangles, 5-ALA PDT; circles, Pd(T4) PDT.

PDT oncolytic activity has been strongly associated with the production of reactive oxygen species (ROS). In Figure 5, ROS production during PDT was assessed visibly via 2’,7’-dichlorodihydrofluorescein diacetate (DCF) staining. C918 cells exposed to PdT4 with 405 nm irradiance exhibited pronounce ROS production compared to cells alone (Figure 5A,B). To further understand the differences in ROS production post-PDT between photosensitizers, ROS levels were quantified in real time in 10 min intervals for the first hour and continued to 6 h. Both photosensitizers demonstrated similar levels of ROS production immediately post-PDT, although Pd(T4) treatment maintained prolonged ROS levels throughout the first hour (Figure 5C).

### 2.5. Macromolecular Interactions with Pd(T4)

**Figure 6:**
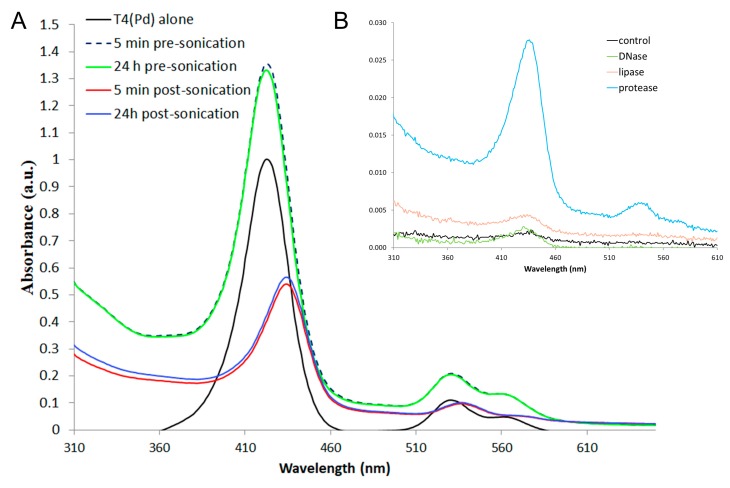
Macromolecule binding of metalloporphyrin Pd(T4). (**A**) C918 cells were incubated with 10 µM of Pd(T4) in a T25 flask for 5 min or 24 h before harvesting the cells. Cells were pelleted and cell-free supernatants as well as porphyrin alone were analyzed by spectrophotometry to determine the absorbance spectrum. To determine porphyrin binding within cells, pellets were resuspended in phosphate buffered saline (PBS) and sonicated followed by centrifugation. Supernatants post-sonication were analyzed to compare bound porphyrin at 5 min compared to 24 h pre-treatment. (**B**) The insoluble pellet was further characterized by being resuspended in PBS and divided into four equal volumes. Each vial was treated differently—PBS alone (control), phospholipase (lipase), DNase, or trypsin (protease)—followed by centrifugation. Absorbance spectrums were determined of supernatants.

Considering the ROS production evident post-Pd(T4) PDT, determining which macromolecules bind to Pd(T4) would suggest targets of PDT. C918 cells were incubated with Pd(T4) for 5 min and 24 h before analyzing the absorbance spectrum. The amount of free photosensitizers in the supernatant was slightly less at 24 h compared to 5 min, which corresponded to an increase (4.7%) in PdT4 binding at 24 h to soluble cellular constituents post-sonication (Figure 6A). This slight difference between pre-illumination incubations and photosensitizer binding mimics the minimal influence on oncolytic activity (Figure 2A). Pd(T4) binding was evident with a shift in the soret peak from free porphyrin at 423 nm to the bound form at 434 nm (Figure 6A). To further assess the macromolecular associations of Pd(T4), the insoluble pellet post-sonication was resuspended with different enzymes individually, namely phospholipase, protease, and DNase. If the porphyrin was associated with macromolecules, after digestion it would be found in the soluble fragment. The majority of Pd(T4) was bound to protein; however, DNA and phospholipid binding was also evident though, at lower levels (Figure 6B).

### 2.6. Pd(T4)-PDT Induces Oncotic Necrosis

**Figure 7:**
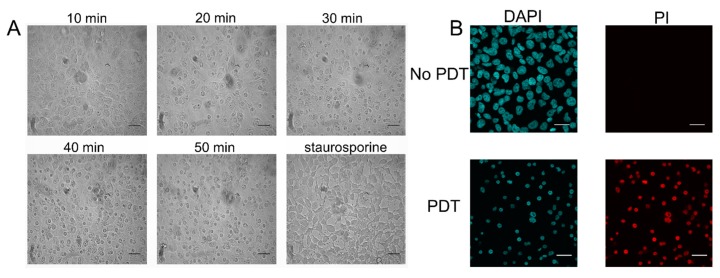
Oncosis responsible for Pd(T4)-PDT oncolytic activity. (**A**) C918 cells post-PDT with 10 µM Pd(T4) and 5 J/cm^2^ of 405 nm LEDs at 10, 20, 30, 40, and 50 min, or exposed to staurosporine (5 µM) after 3 h, representing apoptotic cells. Scale bars equal 100 µm. (**B**) Cells were grown to confluency in 8 well chamber slides and left untreated or treated with 10 µM Pd(T4) followed by 5 J/cm^2^ of 405 nm light and stained with propidium iodide 15 min post-PDT to determine necrosis involvement. Scale bars equal 30 µm. DAPI: 4′,6-diamidino-2-phenylindole; PI: propidium iodide

Morphological analysis of cells treated with PDT using Pd(T4) and 405 nm PDT were analyzed to determine mode of death. Analyzing morphological changes every 10 min through to 50 min with Pd(T4)-PDT compared to 3 h with staurosporine as an apoptosis control highlighted significant differences (Figure 7A). As early as 10 min post-PDT, microtubule and intermediate filaments disruptions are apparent, indicated by cell swelling. This phenotype appears similar to oncosis, which exhibits ionic pump leakage, resulting in cellular swelling, as well as microtubule disorganization. However, during this oncolytic pathway nuclear membranes retain their morphology, unlike staurosporine-induced apoptotic blebbing (Figure 7A). Propidium iodide staining was utilized to determine necrotic pathways or perforated membrane. In Figure 7B, Pd(T4) with 405 nm irradiation exhibited shrunken nuclei positive for propidium iodide 45 min post-PDT supporting a permeable membrane, which was evident in fixed cells as early as 15 min following PDT treatment.

### 2.7. Anticancer Signaling Pathways Modulated by Pd(T4)-PDT Compared to ALA-PDT

**Figure 8:**
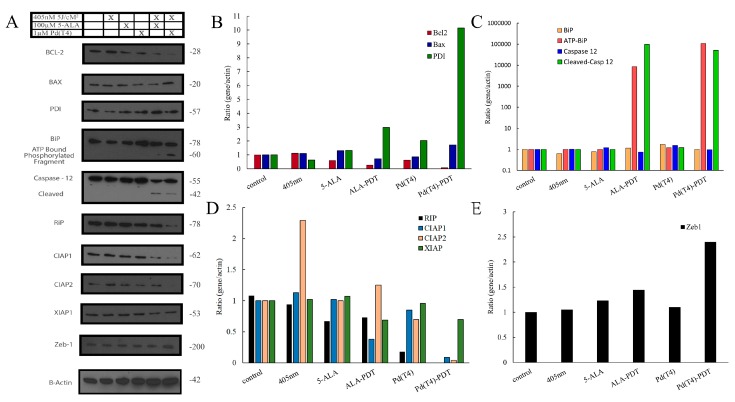
Protein expression in C918 cells exposed to sub-lethal PDT dose. Total protein was isolated from C918 cells alone, exposed to 5-ALA (0.1 mM) or Pd(T4) (1 µM) with or without 5 J/cm^2^ of 405 nm alone.Sodium dodecyl sulfate polyacrylamide gel electrophoresis (SDS-PAGE) was performed followed by Western blot analysis on protein isolated from cells 6 h post-PDT treatment (**A**). ImageJ software was used to quantify protein levels, which were grouped into proteins associated with apoptosis (**B**), endoplasmic stress (**C**), necroptosis (**D**), and other mechanisms (**E**). BiP: binding immunoglobulin protein; RIP: receptor interacting protein; CIAP: cellular inhibitor of apoptosis protein; XIAP: X-linked inhibitor of apoptosis; Zeb1: zinc finger E-box binding homeobox.

To determine the underlying molecular oncolytic mechanisms involved during Pd(T4)-PDT compared to ALA-PDT, C918 cells were stimulated with sub-optimal concentrations of photosensitizers of either 1 µM Pd(T4) or 100 µM 5-ALA. This was performed due to the prompt oncolytic nature of Pd(T4)-PDT at 10 µM. Additionally, considering the three-dimensional nature of target tissues, some cells in the tissue would receive sub-optimal concentrations of photosensitizers. Protein expression was analyzed 6 h post-treatment between 405 nm alone, ALA-PDT, or Pd(T4)-PDT compared to cells alone. The exposure to 405 nm or photosensitizers alone had minimal influences on anti-apoptotic protein Bcl-2 or proapoptotic proteins Bax and protein disulfide isomerase (PDI) (Figure 8A,B). However, Pd(T4)-PDT demonstrated the strongest apoptotic molecular signature, changing the Bax/Bcl-2 ratio from 1.0 (control cells) to 21.5, whereby ALA-PDT exhibited a 2.7 Bax/Bcl-2 ratio (Figure 8A,B).

The influence of PDT on endoplasmic reticulum stress proteins was analyzed using BiP/GRP78, an immunoglobulin protein with chaperone functions. Throughout all treatments, the steady expression of the complete protein was observed (Figure 8A,C); however, the presence of a 60 kDa fragment was only present in PDT treatments with Pd(T4)-PDT cleaving 13.3-fold more BiP than ALA-PDT. Caspase-12, also known for its involvement in ER stress, only displayed the cleaved form amongst cells post-PDT treatments, further supporting the notion of ER disruption.

Another mechanism of cell death is called necroptosis. This pathway involves cellular swelling progressing to rupture of the plasma membrane, which is mediated via receptor-interacting protein kinases (RIPK) [36]. Amongst the different treatments in this study, Pd(T4)-PDT diminished RIP expression the most with only 18% of control cells (Figure 8A,D). The inhibitor of apoptosis protein (IAP) family ubiquitinates RIPK, thereby targeting the necrosome protein complexes for degradation and inhibiting necroptosis [36]. Figure 8A,D display a drastic reduction in both c-IAP1 and c-IAP2 (ratios of 0.09 and 0.04, respectively) after Pd(T4)-PDT, whereby ALA-PDT only reduced c-IAP-1 (ratio of 0.38). The impact of photosensitizers alone had minimal effects, although photo treatment alone doubled the production of c-IAP2 (ratio 2.3). The last of the IAP family analyzed was XIAP-1, which demonstrated similar levels throughout all treatments (Figure 8A,D).

Expression of the zinc finger E-box binding homeobox 1 (ZEB-1) is associated with epithelial-mesenchymal transition in carcinoma cells, and thus is important in assessing if PDT might act as a risk factor for promoting metastasis in cells not killed by PDT [37]. It has also been implicated in chemo- and radio-resistance through its transcriptional regulation of genes involved in DNA damage response [38]. Treatment with Pd(T4)-and ALA-PDT methods resulted in slight increases of Zeb-1 of 2.4 and 1.44, respectively, compared to control cells (Figure 8A,E). Minimal differences were evident once again with photosensitizers or light alone.

## 3. Discussion

This study aimed to demonstrate the functionality of the novel photosensitizer Pd(T4) when used as a treatment modality juxtaposed with irradiance of 405 nm light against an invasive C918 cell line in two- and three-dimensional systems in vitro. Our study demonstrated a stronger effectiveness and utility of Pd(T4) PDT compared to the current FDA-approved PDT photosensitizer 5-ALA, which demonstrates strong success rates against precancerous actinic keratosis and the most common form of skin cancer basal cell carcinoma using either blue or red light [34]. Our findings identify similar oncolytic efficacies of Pd(T4) with 5-ALA when irradiated with 405nm LEDs, but with one hundredth of the concentration and significantly shorter pre-illumination times (5 min vs. 2 h respectively) using Pd(T4). Additionally, vascular mimicry cells were eliminated with Pd(T4)-PDT though 5-ALA-PDT detached cells from the Matrigel in a three-dimensional model. Sub-optimal concentrations of Pd(T4) worked cumulatively with 5-ALA, suggesting future benefits of combinatorial treatments.

Our previous work established that metalloporphyrin Pd(T4) produces significantly more singlet oxygen than its core porphyrin ring TMPyP or metalloporphyrin Zn(T4) when excited by 405 nm LEDs [27]. When utilized as a photosensitizer and illuminated by 405 nm light, increasing Pd(T4) concentrations exhibited more pronounced oncolytic activity, mimicking patterns of TMPyP [31,39]. Conversely, the FDA approved photosensitizer 5-ALA had only a small window of optimal activity (300–1000 µM) before losing some of its lytic activity at higher concentrations. Clinically, the heterogeneity in porphyrin uptake within a tumor could influence the efficacy of treatment, suggesting Pd(T4) might have a clinical advantage, though more in vivo support is needed.

The cellular uptake of porphyrins are driven by different mechanisms. After 5-ALA enters the cell, it is incorporated into the heme biosynthetic pathway, where it is subsequently converted to protoporphyrin IX (PPIX), the active form of the photosensitizer. Cellular localization of PPIX is primarily associated with the mitochondria. Although FDA-approved, the narrow window of active concentrations in our study, increased duration of time required to convert to PPIX, and localization only in the mitochondria suggest potential for improvements. Additionally, the efficacy of 5-ALA in various tumors is also dependent on the activity of the efflux pump ABCG2 [40], thereby being inversely proportional. In our study, Pd(T4) exhibited oncolytic activity as soon as 5 min pre-illumination, which is significantly shorter than previously recorded at 2 h [41]. One of the facets contributing to the efficacy of TMPyP as a photosensitizer can be attributed to its diverse locations within the cell, accumulating primarily in the lysosome [42,43], as well as in the cytoplasm, phospholipid membrane [44], microtubules [31], and the G-rich sequences of DNA and mRNA [41]. The latter has been further characterized by Rapozzi et al., where TMPyP was intercalated with the G-rich 5′-untranslated region of *ras* genes [41] forming a G-quadruplex. Considering these genes are typically overexpressed in cancer cells [45], their subsequent degradation after illumination [41] highlights another benefit of PDT against cancer. Our data further agreed with previous TMPyP findings [41,44,46], where results obtained with DNase and phospholipase (Figure 6) led to the conclusion of a low level of complex formation with DNA and phospholipids, although most of the binding was with proteins. Together, these findings support using both 5-ALA and Pd(T4)-PDT simultaneously to target mitochondrial- and lysosomal-mediated death pathways, potentially leading to synergistic effects.

Most PDT studies examine photosensitizers against cancer cells in vitro in a 2-D model or in vivo. However, even among in vivo experiments, few have analyzed the effects against cancer stem-cell-like cells. When grown on Matrigel, C918 develop a sub-population of cells expressing CD271, a cancer stem cell marker. Efficacy of long-term treatment is dependent on this population. PdT4 exhibited strong oncolytic activity, albeit at higher irradiance doses (15 J/cm^2^) and porphyrin concentrations (100 µM), against C918 epithelial and CD271-positive cells. However, ALA-PDT dislodged the tumor from the Matrigel, suggesting a possible risk of dissemination. After PDT, trypan blue exclusion suggested cells were still viable, although longer monitoring might have demonstrated anoikis.

Due to the divergent pathways of TMPyP and 5-ALA targets within cancer cells, the cumulative effect we demonstrated between sub-optimal concentrations of photosensitizers against C918 cells suggest a new treatment option. Previously, the common photosensitizer zinc(II)-phthalocyanine (ZnPc) also demonstrated a cumulative effect with TMPyP, both in vitro and in vivo [47]. The potential for additive activity with drugs such as Pd(T4) goes beyond that of just other photosensitizers. One potential avenue is to utilize TMPyP4 drugs in conjunction with other therapies that manipulate G-quadruplex structures of DNA and RNA. The potential of this system would be based on the strategies of each individual therapy. Utilizing the effects confirmed within this study along with the ability of TMPyP4, the base of Pd(T4), to distort G-Quadruplex structures [48,49] could induce combinatorial or synergistic effects against cancer cells. Photodynamic destruction is mediated typically by the release of reactive oxygen species. We demonstrated ROS production in ALA-PDT and PdT4-PDT immediately after exposure. Due to porphyrin localization in the nucleus, cytoplasm, lysosome, and membrane, ROS production would damage the cell at multiple foci. However, post-internalization 5-ALA was chosen for our studies given its usage in actinic keratosis with 405 nm light. Treatment times with each drug can vary drastically. Literature commonly links 5-ALA to the incubation range of 2–4 h and with a concentration of 1 mM [19,20,50]. Pd(T4) showcased oncolytic ability with concentrations as low as 10 μM and with incubations times as quick as 5 min, compared to a concentration of 1 mM and incubations times of 2 h for 5-ALA in C918. As previously mentioned, 5-ALA is metabolized by the mitochondria to PPIX, while Pd(T4) is active upon binding affinity.

While observing the oncolytic effects of each drug within our PDT system, the morphologic changes in cells as demise approached were evident. Pd(T4)-treated cells carried traits of necrosis as early as 15 min post-irradiance. The time lapse of propidium iodide (PI) staining in a necrotic system correlates with peak generation of ROS [51]. The usage of H_2_O_2_ in this study displayed characteristics of necrosis, even when void of the presence of RIP1. These characteristics are akin to what we have seen with treatment of Pd(T4)- PDT. The downregulation of RIP1 coinciding with the disruption of the plasma membrane allowed PI staining to completely stain the remnants of the nucleus, further supporting necrosis as the modality of death following Pd(T4)-PDT.

To determine the underlying effects of Pd(T4) and 5-ALA-PDT, key proteins involved in cell death pathways were explored, utilizing sub-inhibitory concentrations of each treatment. Sub-lethal concentrations were utilized due to the binding affinity of Pd(T4) to proteins, as well as the prompt onset of necrosis. Bcl-2 is an anti-apoptotic marker commonly implicated in the inhibition of apoptosis [52]. When combined with the upregulation of Bax, cell death ensues via mitochondrial outer membrane permeabilization (MOMP), facilitating cytochrome c release upstream of caspase activation [53]. This ratio of Bax/Bcl-2 is linked to how sensitive or resistant cancer cells are to treatments [54]. We observed a substantial shift towards a greater Bax/Bcl-2 ratio in Pd(T4)-PDT. Additionally, Pd(T4)-PDT upregulated endoplasmic reticulum chaperone protein disulfide isomerase (PDI), which also facilitates MOMP via the oligomerization of BAK in replacement of Bax [55]. This enables MOMP to release cytochrome c and other mitochondrial proteins in a dose-dependent manner beyond functional roles in the ER and other organelles [56,57]. The activity of PDI has been linked to interactions with ubiquilin [58], thus allowing the uninhibited lysosomal protein degradation [59]. GRP78/BiP is another pivotal protein in the chaperone function [60,61] of the ER that was analyzed. The preservation of the observed 60 KDa fragment by the PDT-treated cells was indicative of degradation of functional GRP78/BiP, but also of the presence of ATP [61] displaying priming of oncolytic activity. ER stress is further hallmarked in our study by the presence of cleaved caspase 12, seen by the auxiliary band [62] present in the PDT-treated variables.

The repression of RIP along with that of the IAP family is indicative of the dissociation of the TNF-α/TNFR1 complex 1, which regulates the necroptosis pathway [63]. The decline in the presence of this complex not only has a potential positive impact on the inflammation response that a host would have in response to treatment [63], but also decreases the likelihood of chemoresistance developing through the inhibition of the IAP family of proteins [64]. Downregulation observed of both CIAP1/2 and RIP1 does confirm the eventual absence of TNFα-induced NF-κB response [65]. The observed downregulation of the IAP family and RIP1 in the Pd(T4) model exhibits the temporal nature of PDT-elicited activity. CIAP 1/2 is known to act as a ubiquitin ligase inhibiting RIP1 through proteosomal degradation [66]. This activity seems to happen before the IAP family is complexed with Smac [67], following its release from the mitochondria via MOMP induced by PDI and Bax.

Zeb1 has previously been found to be present in C918 cell lines, driving cellular proliferation through the repression of cyclin-dependent kinase inhibitors and tumor suppressor genes, as well as promoting tissue invasion and metastases [68]. However, other cancer cell line studies linked Zeb1 with transcriptional regulation of DNA damage response genes [38,69]. In our study, PDT upregulated Zeb-1 compared to porphyrin or light alone. Due to Pd(T4) binding to DNA (Figure 6B), this suggests Zeb1 expression is associated with clearing DNA double-strand breaks through checkpoint kinase 1 [69]. Considering chemoresistance and vasculogenic mimicry formation are associated with Zeb1 expression, further studies are needed to elucidate the role of Zeb1 in response to PDT. Additionally, our findings of Pd(T4)-PDT exhibiting oncolytic activity against vascular mimicry cells supports its use in combination with other chemotherapeutic agents.

As PDT progresses as a growing field into the future, advancements to improve the clinical efficacy of treatments will be the cornerstone to the development of PDT. Overcoming the hurdle of light penetrance into tissue is at the forefront of the field. Methods to overcome this hurdle include the use of techniques in unique ways. The utilization of fluorescence microscopy techniques, such as two-photon imaging, can increase the penetrance and planar-specific excitation of photosensitizers that accumulate in deeper tissues but lack a near-infrared (NIR) absorbance spectrum. One particular example that shows promise for the utilization of photosensitizers and other light-activated reagents that excite outside of the NIR range used two-photon NIR. For example, blebbistatin when conditioned with blue light undergoes photoexcitation, resulting in hydroxide radicals and subsequent cytotoxicity, even in hypoxic environments. Although lacking absorption outside the blue spectrum, when irradiated with 800 nm using a two-photon NIR, blebbistatin exhibited similar effects [70]. This display of transition from a one-photon blue light method to a two-photon NIR is a display of development that would unlock the clinical efficacy of blue light photosensitizers. Juxtaposed with the usage of two-photon NIR excitation of blue light photosensitizers, another fluorescent microscopy technique is being adjusted for use in the PDT field is Förster resonance energy transfer (FRET) [71]. A study used FRET to ultimately energize TMPyP through two-photon NIR excitation of a carbon dot conjugated to TMPyP [72]. This “up-conversion” method would be an exciting future direction for Pd(T4) [71]. Aptamer conjugations to TMPyP have also been conducted to increase the tumor-targeted delivery of TMPyP PDT [73]. The exploration and development of contemporary practices such as the ones mentioned and those yet to be discovered will help usher PDT further into the future. Using these advancements with Pd(T4) might help the clinical efficacies of treatment.

## 4. Materials and Methods

### 4.1. Cells and Media

Uveal melanoma cell line C918, derived from primary uveal melanoma and characterized by Folberg et al. [74], was gifted from Dr. R. Folberg and maintained in Dulbecco’s modified Eagle’s media (DMEM) (Corning, Tewksbury, MA, USA) supplemented with 10% (*v/v*) fetal bovine serum (Atlanta Biologicals, Minneapolis, MN, USA), 100 U/mL penicillin, 100 µg/mL gentamycin, 0.25 µg/mL of amphotericin B (Sigma-Aldrich, St. Louis, MO, USA), and 2 mM L-glutamine (Gibco, Gaithersburg, MD, USA) (complete (C)-DMEM). Cultures were maintained in a humidified incubator at 37° C with 5% CO_2_.

### 4.2. Synthesis of Pd(T4) Photosensitizer

The water soluble chromophore (5,10,15,20-tetrakis-(N-methylpyridynium-4-yl)porphyrin)palladium(II) was synthesized following published procedures [75]. Briefly, to synthesize an aqueous solution containing (H_2_(T4)) (NO_3_)_4_ (Sigma-Aldrich), a ten-fold molar excess of Pd(DMSO)_2_(H_2_O)_2_^+2^ was added in three aliquots and refluxed for 48 h, during which the reaction progress was monitored via absorption. Upon completion of the reaction and filtration, addition of KPF_6_ in acetonitrile to the filtrate precipitated the isolated product. Ion exchange with tetrabutylammonium nitrate yielded the water-soluble nitrate product.

### 4.3. Cell Culture and Photodynamic Therapy

C918 cells were seeded in 6-well plates with 2 × 10^5^ cells/mL and incubated for 48 h at 37 °C and 5% CO_2_, reaching 90–100% confluency. Photodynamic treatments were performed analyzing various concentrations of Pd(T4) or 5-ALA (Sigma-Aldrich) at different times, prior to varying intensities of 405 nm irradiation from a portable LED emitting 88 s pulses of 60 mW/cm^2^ producing 5 J/cm^2^ (WARP, Quantum Devices, Barneveld, WI, USA). For combinatorial studies with both photosensitizers, cultured cells were incubated with 5-ALA 5 min before Pd(T4) addition. Wells were exposed to 405 nm light at various doses subsequent to a 2 h incubation with Pd(T4).

For three-dimensional culture, 250 µL of Matrigel^®^ (Corning, Corning, NY, USA) was distributed confluently over the surface of a well in a 6-well plate. Polymerization occurred after one hour at 37 °C and 5% CO_2_. Wells were washed with C-DMEM and subsequently seeded with 2 mL of 2 × 10^5^ cells/mL and incubated at 37 °C and 5% CO_2_ with media change every two days. After 120 h, photosensitizers were applied for 2 h before irradiation with 405 nm light. Following treatment, plates were placed at 37 °C and 5% CO_2_ for 24 h before trypan blue viability tests.

### 4.4. Assessment of Cell Viability Using MTT Reduction

MTT assays were performed to determine viability. Briefly, 50 µL of 5 mg/mL of 3-(4,5-dimethylthiazol-2-yl)-2,5-diphenyltetrazolium bromide (MTT) (Sigma-Aldrich) was added to wells for 3 h with a subsequent Hank’s balanced salt solution (HBSS) wash. The resulting crystallization was solubilized in 750 µL of 90% isopropanol and 10% Triton X-100 at a pH of 4.5. Samples were then transferred to a 96-well plate in duplicates and absorption measurements at 570 nm minus 630 nm background were acquired using an ELx800 absorbance microplate reader (Biotek, Winooski, VT, USA) with Gen5 Software. MTT reduction was used to assess cell viability and calculated using the following equation, where control cells were incubated with media alone.

MTT reduction (%) = Avg. optical density (OD) of treated group × 100 Avg. OD of control cells

Three-dimensional culture viability was analyzed using trypan blue exclusion assay. Briefly, media was aspirated and incubated for 10 min with 0.2% trypan blue (Sigma-Aldrich). Cells were counted in triplicates of 100 for each well to analyze three different cell populations: vasculogenic mimicry-forming cells, cells growing on the top of Matrigel, and cells growing on the bottom of the wells.

### 4.5. ROS Detection Assay

C918 cells were plated and grown to 100% confluency in an eight-chamber well slide at 37 °C and 5% CO_2_. Once confluent, 10 µM Pd(T4) or 1 mM 5-ALA was added and incubated for 2 h, followed by irradiance with 5 J/cm^2^ of 405 nm light. Subsequent to PDT, each well was cultured with 10 μM of ROS immunofluorescence dye (2′,7′ dichlorofluorescein diacetate) (Sigma-Aldrich) and incubated at 37 °C and 5% CO_2_ for 30 min. Immunofluorescence was detected by an Olympus IX73 microscope (Olympus, Tokyo, Japan).

To analyze ROS kinetics, cells were added at 4 × 10^5^ cells per well in a 6-well plate. Photosensitizers were added after 100% confluency and 2 h before light treatment, whereby 5 µM of CellRox™ deep red (Invitrogen, Carlsbad, CA, USA) was added 30 min before irradiance with 5 J/cm^2^ of 405 nm. After light treatment, fluorescence was quantified using live imaging with the IncuCyte^®^ at four separate sampling sites within each well for the first 6 h.

### 4.6. Propidium Iodide Staining

PDT-induced necrosis was assessed in an eight-chamber well slide. C918 cells were plated and grown to 100% confluency. Wells were cultured with or without photosensitizer followed by a 2 h incubation prior to 405 nm light exposure. Following irradiation, samples were treated with 2 µg/mL of propidium iodide (Sigma-Aldrich) and incubated for 5 min. Samples was washed with PBS and fixed with 4% paraformaldehyde for 15 min with subsequent PBS washes, before an incubation with 50 µg/mL of RNase (Qiagen, Hilden, Germany) for 15 min at 37 °C with 5% CO_2._ After a final PBS wash, images were acquired using an Olympus Fv10i Fluoview confocal microscope (Olympus).

### 4.7. Molecular Interactions with Pd(T4)

Cells were plated in a T25 flask and grown to a 100% confluency. To determine photosensitizer binding, 10 µM of Pd(T4) was incubated for 2 h in a 37 °C incubator with 5% CO_2_. Cells were scraped and pelleted at 300× *g* for 10 min and supernatants were collected for later use. The pellets were washed and resuspended with PBS followed by sonication for 20 s at 30 watts using the F60 Sonic Dismembrator (Fisher Scientific, Hampton, NH, USA). After sonication, each sample was centrifuged at 8160× *g* for 10 min. Pellets were resuspended in PBS and divided into four treatment groups to determine Pd(T4) molecular associations: 1—control; 2—DNase (1 mg/mL) (Sigma-Aldrich); 3—phospholipase C (12.5 U) (Sigma-Aldrich); 4—trypsin (50 µg/mL). After 37 °C overnight incubation, samples were centrifuged at 8160× *g* for 10 min and absorption spectra (250–650 nm) were determined for supernatants.

### 4.8. SDS-PAGE and Western Blotting

Molecular signaling of cell death was analyzed by Western blot, though due to prompt cell death at 10 µM, sub-lethal concentrations of photosensitizers were used. Briefly, wells were washed 6 h post-treatment and disrupted using radio immunoprecipitation assay (RIPA) lysis and extraction buffer (Thermo Fisher Scientific, Waltham, MA, USA) with a proteinase inhibitor cocktail (Roche, Basel, Switzerland). Cells were further scraped, collected, and placed on ice. Protein concentrations were quantified using bicinchoninic acid (BCA) assay (Pierce, Waltham, MA, USA) and 50 µg was loaded per lane in a 6% or 12% Tris-glycine SDS-PAGE gel. After electrophoresis, proteins were transferred onto a nitrocellulose membrane using a semi-dry transfer apparatus (Bio-Rad Laboratories, Hercules, CA, USA). The membrane was blocked with 5% non-fat skim milk, and further washed with Tris-buffered saline (TBS) + Tween 20 (TBST). Membranes were incubated overnight at 4 °C with various primary antibodies (Cell Signaling Technologies, Danvers, MA, USA, unless otherwise noted): mouse anti-human Bcl-2, rabbit anti-human Bax, rabbit anti-human PDI, rabbit anti-human Bip, rabbit anti-human caspase-12, rabbit anti-human RIP1, rabbit anti-human cIAP-1, rabbit anti-human anti-cIAP-2, rabbit anti-human XIAP, rabbit anti-human TDF-8/Zeb-1, or horseradish peroxidase (HRP)-conjugated mouse anti-human beta actin (Sigma-Aldrich). Membranes were washed with TBST before incubation with HRP-conjugated secondary antibodies (Cell Signaling Technology). For quantification, SuperSignal™ West Pico PLUS chemiluminescent substrate reagent (Thermofisher) was applied to the membrane and exposed to High Speed Blue X-ray film (ScripHessco, Bolingbrook, IL, USA) in a dark room. The band intensities were quantified using ImageJ software.

### 4.9. Statistics

Statistical analysis was performed using GraphPad PRISM 7 software (GraphPad Sofrtware, San Diego, CA, USA). Analysis between different treatment groups and time points was performed using a one-way analysis of variance (ANOVA) with Tukey’s multiple comparison test.

## 5. Conclusions

Pd(T4) has shown significant effects in a PDT system utilizing a porTable 405 nm LED against C918 cells, a highly invasive aggressive epithelioid. The similar oncolytic effects of Pd(T4)-PDT compared to ALA-PDT, although at shorter pre-illumination times (5 min) and lower concentrations (10 µM), suggest Pd(T4)-PDT can reduce treatment durations, including for skin disorders such as actinic keratosis and basal cell carcinoma, thereby decreasing health care costs and patient stress responses. Additionally, the combinatorial effect demonstrated in this study with 5-ALA and PdT4 with 405 nm irradiance might increase the efficacy and diversity of PDT clinical applications, as well as with already existing chemotherapeutic drugs [76]. The oncolytic effects of Pd(T4) against stem-cell-like vascular mimicry cells warrants new approaches using combinatorial PDT treatments with current therapeutic approaches.

## Figures and Tables

**Table 1:** Three-dimesional oncolytic effect of PDT with Pd(T4) and 405 nm irradiance on C918 cells.

Treatment	Dosage (J/cm^2^)	% death in 3D culture		
		Top of Matrigel	VM	Bottom of Plate
No PDT	0	<3%	<3%	<3%
100 M Pd(T4)	0	<3%	<3%	<3%
30 M Pd(T4)	10	5%	<3%	<3%
30mM Pd(T4)	15	5%	<3%	40%
100 M Pd(T4)	10	20%	<3%	50%
100 M Pd(T4)	15	60%	50%	90%

Data is representative of one independent experiment where cells were counted in triplicates of 100 for each well; VM: vasculogenic mimicry-forming cells; PDT was via 405 nm LED.
